# Direct Oral Anticoagulants Are Comparable to Low Molecular Weight Heparin at Sustaining the Circulating Extracellular Vesicle and Inflammatory Profiles of Cancer Associated Thrombosis Patients: An Observational Pilot Study

**DOI:** 10.1002/cam4.70920

**Published:** 2025-04-28

**Authors:** H. Macleod, N. Copty, D. Doherty, L. Weiss, E. Fouhy, R. Power, N. Ryan, K. Saeed, E. ORourke, R. Faryal, S. Kelliher, B. Kevane, F. Ní Áinle, P. B. Maguire

**Affiliations:** ^1^ UCD Conway SPHERE Research Group Conway Institute, University College Dublin Dublin Ireland; ^2^ School of Biomolecular and Biomedical Science University College Dublin Dublin Ireland; ^3^ Department of Oncology Mater Misericordiae University Hospital Dublin Ireland; ^4^ School of Medicine Trinity College Dublin Dublin Ireland; ^5^ Department of Haematology St James's Hospital Dublin Ireland; ^6^ Department of Haematology Mater Misericordiae University Hospital Dublin Ireland; ^7^ School of Medicine University College Dublin Dublin Ireland; ^8^ UCD Institute for Discovery O'Brien Centre for Science, University College Dublin Dublin Ireland

**Keywords:** anticoagulation, extracellular vesicles, inflammation, thrombosis

## Abstract

**Introduction:**

Cancer patients face a 4 to 7‐fold higher risk of developing thrombotic events compared to individuals without cancer. This elevated risk is driven by the underlying tumour biology and the effects of cancer treatments, significantly increasing the mortality rates of these patients. While low molecular weight heparin (LMWH) is the gold standard anticoagulation, direct oral anticoagulants (DOACs) are emerging as effective alternatives. Recent clinical evidence indicates reduced recurrent VTE upon DOAC treatment compared to LMWH; however, there is limited understanding of the underlying mechanistic pathways. Of interest, extracellular vesicles (EVs), released from a multitude of cells including platelets and tumour cells, are known as potent intercellular communication mediators, capable of progressing coagulation, thrombosis, as well as tumour growth and metastasis.

**Methods:**

We characterised the extracellular vesicles and inflammatory markers associated with hypercoagulability and thrombosis in cancer‐associated thrombosis (CAT) patients, comparing those treated for 8 weeks with DOACs to those receiving LMWH. This pilot observational study recruited 28 CAT patients (21 baseline, 13 treated with DOACs, 8 treated with LMWH; 14 paired) and quantified their circulating, platelet‐derived, and endothelial‐derived EVs using Nanoparticle Tracking Analysis and flow cytometry. Proteomics was performed on the EV cargo and patient plasma, quantifying the inflammatory profiles of the patients under both treatment arms.

**Results and Discussion:**

We demonstrated that DOAC treatment maintained hypercoagulable and prothrombotic EV profiles similar to LMWH treatment, showing a remarkably stable EV cargo proteome. Inflammatory profiles were also comparable between treatment arms, with a trend toward a DOAC‐mediated reduction of circulating cytokines, highlighting potential anti‐inflammatory effects.

**Conclusion:**

This pilot study demonstrates that DOACs sustain the circulating EV and inflammatory profiles to the same extent as LMWH, supporting this clinical shift in anticoagulant treatment in the cancer setting.

## 1 Introduction

Cancer associated thrombosis (CAT) is the second leading cause of death among the cancer population after progression of the disease itself [1]. The cancer disease state can tip the delicate balance of thrombosis and haemostasis, resulting in immuno‐thrombosis and potentially fatal thrombotic events. In fact, there is a 4‐to‐7‐fold increase in the risk of cancer patients presenting with a venous thromboembolism (VTE) compared to those without cancer [2] with the annual incidence of VTE rising from 0.1% in the normal population to 0.5% in a cancer setting [3]. Moreover, patients with CAT have a 3‐ to 5‐fold higher risk of mortality compared to cancer patients without thrombotic events [4]. As a result, extensive research over the past decade has focused on developing novel anticoagulant therapies to effectively prevent and manage these thrombotic events.

Low Molecular Weight Heparin (LMWH) emerged as the definitive gold standard for treating CAT following the landmark CANTHANOX and pivotal CLOT trials at the turn of the century, highlighting that LMWH treatment significantly reduced VTE recurrence in cancer patients compared to vitamin K antagonists, with no difference in major bleeding rates [5, 6]. These studies reshaped the therapeutic landscape for LMWH to become first‐line therapy for CAT treatment, with similar results echoed in the CATCH trial a decade later [7]. In 2009, direct oral anticoagulants (DOACs) were introduced for the treatment of VTE, and the first randomised controlled trial, the Hokusai VTE Cancer trial, evaluating the use of DOACs in a cancer‐VTE setting was published in 2018 [8]. This was soon followed by the Caravaggio and SELECT‐D trials [9, 10].

The Hokusai VTE Cancer trial compared edoxaban to dalteparin, showing 3.4% lower VTE recurrence in the edoxaban arm; however, there was a 2.9% increased rate of major bleeding [8]. Similarly, the SELECT‐D trial showed lower VTE incidence with rivaroxaban compared to dalteparin (4% vs. 11%) but again, higher clinically relevant non‐major bleeding (CRNMB) rates were associated with DOAC treatment (13% vs. 4%) [9]. The more recent Caravaggio trial echoed such findings, showing a reduction in VTE recurrences with apixaban compared to dalteparin treatment of CAT patients (5.6% vs. 7.9%; HR: 0.63; CI: 0.37–1.07, *p* < 0.001) [10]. These trials highlight the efficacy and non‐inferiority of DOACs compared to the traditional LMWH treatment in this CAT patient cohort. Multiple meta‐analyses support these findings, of which Michalopoulou et al. showed a 40% reduction in recurrent VTE upon DOAC treatment compared to VKA and LMWH anticoagulant upon systemic review of 8 studies including > 4000 patients, highlighting that the net clinical benefit of DOAC treatment is similar to or more favorable than usual anticoagulant [11, 12, 13]. This led major international guidelines to recommend DOACs for treating cancer‐associated thrombosis, except in high‐risk bleeding cases such as gastrointestinal malignancies [14, 15, 16].

These studies have driven the recent shift from LMWH to DOACs for VTE treatment in cancer patients. However, there is limited understanding of how these anticoagulants impact underlying hypercoagulable mechanisms. Elucidating such effects on these unexpected mechanisms, which are capable of catalyzing future adverse outcomes, would be clinically reassuring and highly relevant.

**FIGURE 1:**
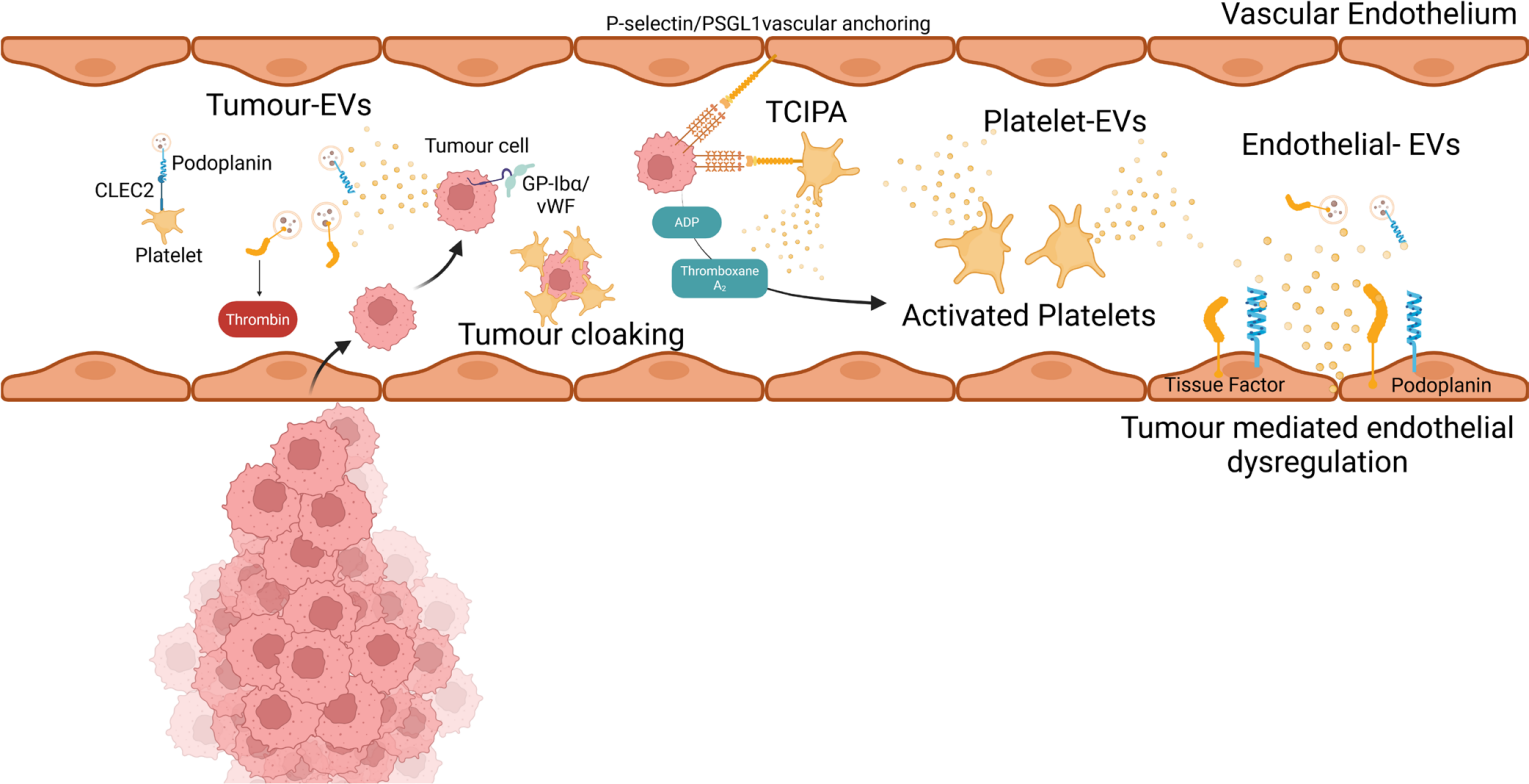
Platelet, tumour and endothelial EV interactions. Tumour cells enter circulation, releasing tumour‐EVs expressing tissue factor and podoplanin, known to initiate the coagulation cascade and cause strong platelet activation. Tumour cells can also bind directly to platelets through the P‐selectin/PSGL‐1 axis in a process called tumour cell induced platelet activation (TCIPA), facilitating tumour cloaking, protecting the tumour cells within circulation. Subsequently, activated platelets release EVs which can be taken up by platelets, tumour cells and the vascular endothelium. The hypoxic and dysregulated tumour microenvironment causes endothelial dysregulation leading to the release of endothelial‐derived EVs, which work in synergy with tumour‐and platelet‐derived EVs, promoting a hypercoagulable and prothrombotic environment to further promote tumour progression.

One potential mechanism could involve a differential effect of DOACs compared with LMWH on extracellular vesicles (EVs) in patients with cancer‐associated VTE [17, 18, 19, 20, 21]. EVs are composed of a lipid bilayer, which can bleb off the plasma membrane forming large EVs or are released from internal multivesicular bodies forming small EVs, influencing the physiological processes upon endocytosis of transported cargo [22]. EVs, released by activated platelets, endothelial cells, and tumour cells, promote hypercoagulability through the expression of platelet receptors and hypercoagulable markers as well as the release of soluble agonists (Figure 1) [23, 24, 25, 26, 27, 28, 29]. Tumour cells can activate platelets and release EVs that in turn promote thrombosis, contributing to cancer progression [30]. Tumour cells release platelet agonists such as ADP and Thromboxane A_2_, along with other potent mediators, known to cause strong and sustained platelet activation [31, 32]. Tumour cells have also been shown to express receptors associated with platelets including αIIbβ3, αVβ3, and GP‐Ibα, promoting thrombus formation [33, 34, 35]. These released platelet EVs can promote thrombosis through multiple mechanisms including positive feedback platelets activation loops, binding and activating neutrophils leading to neutrophil extracellular trap (NET) release and intercellular communication with immune cells [23]. Furthermore, tumour and dysregulated endothelial‐derived EVs expressing tissue factor initiate the extrinsic pathway of the coagulation cascade generating thrombin along with podoplanin, which binds to CLEC‐2 on platelets initiating an activation response [20, 21, 36]. Such platelet, endothelial, and tumour EVs are highly procoagulant, expressing tissue factor, phospholipids, platelet receptors, and internalized soluble mediators, which create a hypercoagulable environment within these patients (Figure 1).

These processes are also intrinsically linked with cancer progression [23]. Activated platelets bind to tumour cells “cloaking” them within circulation, allowing the tumour cell to avoid immune detection, withstand high sheer vascular forces, and aid tumour migration and metastasis [37]. Tumour cells can express the essential P‐selectin Glycoprotein Ligand‐1 (PSGL1) which binds to P‐selectin on platelets and endothelial cells (as well as their derived EVs) anchoring the tumour cell to the vascular endothelial, aiding migration and metastasis [38]. Tumour cells in circulation lead to EV release, which aids cancer development and thrombus formation, encompassing an essential mechanism to investigate in this patient cohort (Figure 1).

In addition to prothrombotic EVs, the heightened inflammatory state of cancer is known to cause endothelial dysregulation, platelet activation, decreased fibrinolysis, and activation of the coagulation cascade, all processes tightly linked to thrombosis formation and cancer progression [25, 39, 40]. Recent evidence has revealed anti‐inflammatory properties of both DOAC and LMWHs, an intriguing pleiotropic effect of which could be essential in the treatment of cancer‐associated thrombosis [41, 42, 43]. Therefore, we aimed to understand the inflammatory profiles of CAT patients treated with DOACs compared to LMWHs, to compare such anti‐inflammatory potential.

Platelet activation, EV signalling, inflammation, and cancer progression are deeply intertwined; therefore, it is essential to investigate whether anticoagulants, particularly DOACs, influence these EV‐and inflammatory‐driven processes in unexpected ways, to provide reassurance to treating clinicians beyond the already advantageous VTE and bleeding rates. Current data suggest that DOACs do not adversely impact these pathways compared to LMWHs, with some evidence pointing to greater effects [41], it is important to confirm this in CAT patients differentially treated with LMWH and DOACs.

## 2 Material and Methods

### 2.1 Patient Eligibility and Enrolment

Patients were recruited to the EXPECT Study following ethical approval granted by the Research and Ethics Committee Board in the Mater Misericordiae University Hospital (MMUH: Ref 1/378/2169: 21/03/2022), with an approved amendment to add an additional timepoint sample on 16/02/2023. Patients with active cancer who were diagnosed with a VTE event and treated with either DOAC or LMWH at the MMUH were approached to participate in the EXPECT Study, provided they met the inclusion and exclusion criteria. Following informed written consent according to the declaration of Helsinki, a baseline sodium citrate platelet poor plasma (PPP) blood sample was obtained followed by a follow‐up sample upon 2–9 weeks on anticoagulant treatment. Patient data was also collected for study analysis.

### 2.2 Blood Collection and Processing

Blood samples for the EXPECT Study were collected in sodium citrate vacutainers (0.106 M final concentration; BD Vacutainer, Franklin Lakes, New Jersey, USA) and immediately sent to the MMUH Haematology laboratory for processing. Whole blood samples were centrifuged at 1811 × *g* for 6 min at room temperature to isolate the platelet poor plasma (PPP), which was separated and centrifuged at the same speed/time/temperature to remove any platelet contamination. PPP was frozen at −80°C until thawed for downstream analysis.

### 2.3 Nanoparticle Tracking Analysis (NTA)

Particle size distribution in PPP was determined by NTA using a NanoSight NS300 system (Malvern Technologies, Malvern, UK) fitted with a 488‐nm laser and a high‐sensitivity scientific camera, as previously described [44]. Plasma was diluted (1:100–1:2000) in particle‐free phosphate‐buffered saline (PBS; Gibco, Waltham, Massachusetts, USA) to achieve 10 to 80 particles per frame, according to the manufacturer's instructions. Sample analysis was performed at 25°C and a constant flow rate of 50 (arbitrary units) [44]. The 15 × 60 s videos were captured with a camera level of 13, and data were analysed using NTA 3.1.54 software with a detection threshold of 10, as documented before [44].

### 2.4 Flow Cytometry

#### 2.4.1 EV Counting Calibrated With Polystyrene Beads

Flow cytometry analysis of circulating EVs was performed using a CytoFlex S (Beckman Coulter, Brea, USA), with particle size calibrated using commercially available polystyrene beads. 30 μL platelet‐poor plasma (PPP) was diluted with 520 μL 0.22 μm filtered PBS. Samples were further diluted in PBS for optimal counting conditions and to prevent EV swarming (1:20–1:1000) and analyzed in triplicate at a constant flow rate of 10 μL/min for 2 min or until 100,000 events were recorded. Polystyrene beads are solid spheres with a refractive index of 1.61 [45], whereas vesicles consist of a core and a shell with refractive indices of 1.38 and 1.48, respectively. Compensation for differences in refractive indices was performed using Rosetta calibration beads (Exometry, Amsterdam, Netherlands) and the Rosetta calibration software (version 1_30) [45]. This calibration is based on the Mie theory and used to relate side scatter intensities (in arbitrary units) to EV diameter (in nm) for a given refractive index [46]. Data analysis was performed using Kaluza software (version 2.1, Beckman Coulter). Resulting data was analysed using RStudio (version 4.2.1).

#### 2.4.2 EV Immunofluorescent Staining

For EV immunofluorescent staining of platelet and endothelial markers, EVs were isolated from 100 μL of PPP by centrifugation at 20,800 × *g*, for 10 min at 10°C. Leaving 10 μL behind, the EV pellet was washed with 1 mL of 0.22‐μm filtered PBS (Gibco) and centrifuged at 20,800 × *g*, for 10 min at 10°C, leaving 20 μL behind. The washed EV pellet was resuspended in 230 μL Annexin‐V Binding Buffer (BD Pharmingen, San Diego, USA). 50 μL of this EV suspension was separated into four tubes (unstained, Annexin‐V only, platelet markers‐CD41, CD42b, CD62P, Annexin‐V and endothelial markers‐ CD31, CD142, podoplanin, Annexin‐V) and stained at room temperature for 20 min in the dark with corresponding fluorophore‐conjugated antibodies; Annexin‐V‐FITC (1 μL of antibody used), CD41‐PB, CD42b‐APC, CD62P‐PE, CD31‐PB, CD142‐APC, podoplanin‐PE (2.5 μL of antibody used) (BioLegend, San Diego, USA). Excess antibody was washed off with 300 μL Annexin‐V binding buffer, centrifuged at 20,800 × *g*, for 10 min at 10°C, leaving 20 μL behind. The final stained EV pellet was resuspended in 200 μL Annexin‐V binding buffer for flow cytometry analysis. Samples were analyzed at a constant flow rate of 10 μL/min for a stable time of 2 min, with an Annexin‐V binding buffer wash step between tubes and samples. Gating was performed on the CytExpert software (version 1.0, Beckman Coulter, Brea, USA). Annexin‐V‐FITC staining was gated upon the stable time events, with this gate used to gate corresponding markers for double positive staining. Unstained and Annexin‐V only staining tubes ensured accurately defined gates. Data was expressed in events and corrected based on the dilutions used to determine the events per μL plasma. Resulting data was analyzed using RStudio (version 4.2.1).

#### 2.4.3 Sucrose Cushion Ultracentrifugation

EVs were enriched from PPP with sucrose cushion ultracentrifugation for downstream tandem mass spectrometry and western blot analysis (as previously described [41]). 100 μL PPP was diluted with 850 μL of filtered, ice‐cold particle‐free PBS (Gibco) and centrifuged at 3000 × *g* for 5 min at room temperature to pellet debris. Supernatant was transferred to an ultracentrifuge tube (Beckman Coulter, Brea, USA) where it was layered onto 50 μL of a 30% sucrose/198 mM Tris/D_2_O cushion (pH 7.4), without disturbing the interphase. Samples were centrifuged at 52,000 rpm corresponding to ~120,000 × *g*, 4°C for 6 h (hours) in a Beckman Coulter Optima MAX‐XP Ultracentrifuge fitted with an MLA‐130 rotor, ensuring equal weight of all tubes. Supernatant was discarded and EV/sucrose cushion was transferred to a new tube and frozen at −80°C.

### 2.5 Mass Spectrometry

#### 2.5.1 Sample Preparation: In Solution Double Digest With Commercial PreOmics Kit and EvoTip Loading

EVs were enriched via sucrose cushion ultracentrifugation and prepared for mass spectrometry analysis using the iST Sample Preparation Kit 192x, according to the PreOmics protocol, as previously described [47] (PREOMICS, Martinsried, Germany). In short, 200 μg of enriched EV proteins were lysed with the PreOmics LYSE buffer on a 95°C heating block, 1000 rpm, for 10 min. With the cartridge placed on top of the waste in the adapter plate, the samples were transferred and allowed to cool before the DIGEST buffer was added. The double digest with LysC and trypsin was allowed to incubate at 37°C, 500 rpm, for 1 h. The STOP buffer was added to the cartridge and centrifuged at 2250 × *g* for 2 min to collect flow‐through. Samples were washed twice with two wash buffers, repeating this centrifugation step. Using the MTP plate, the ELUTE buffer was added to the cartridge, repeating this centrifugation to elute peptides from the column. This elution step is repeated twice. Peptides were collected in the MTP plate and placed in a vacuum evaporator at 45°C until completely dry. LC‐LOAD was added to the MTP plate, centrifuged at room temperature for 5 min at 500 rpm, and peptides were transferred to low‐bind tubes. Nanodrop at 280 nm allowed for peptide concentration to be determined to bring concentration to 2.5 μg peptides in a total volume of 50 μ with LC LOAD (0.05 μg/μL final concentration).

Complete purification and sample preparation of the peptides was performed using Evotips (EVOSEP, Buchwaldsgate, Denmark). Each Evotip was activated with 25 μL of methanol, centrifuged at 700 × *g* for 1 min, collecting the flowthrough. 25 μL of solvent A (99.9% water and 0.1% formic acid) was added to each tip and centrifuged as indicated. The maximum loading capacity of the Evotip of 1 μg was loaded (20 μL of the 0.05 μg/μL peptide sample) and added to each Evotip and centrifuged again as indicated. Lastly, 120 μL of solvent A was added to each tip to ensure tips remained wet and stored at 4°C before being placed on the Evosep One LC System.

#### 2.5.2 Liquid Chromatography Tandem Mass Spectrometry Analysis

Proteomics samples were loaded onto individual EvoTips and run on a timsTOF Pro mass spectrometer (Bruker Daltonics, Bremen, Germany) coupled to the EvoSep One system as previously described [47] (EvoSep BioSystems, Odense, Denmark). The peptides were separated on a reversed‐phase C 18 Endurance column (15 cm × 150 μm ID, C 18, 1.9 μm) using the preset 30 SPD method. Mobile phases were 0.1% (v/v) formic acid in water (phase A) and 0.1% (v/v) formic acid in acetonitrile (phase B). The peptides were separated by an increasing gradient of mobile phase B for 44 min using a flow rate of 0.5 μL/min.

Data Dependent Acquisition (DDA) was run on all samples to build a spectral library for Data Independent Acquisition (DIA). For DDA, the timsTOF Pro mass spectrometer was operated in positive ion polarity with Trapped Ion Mobility Spectrometry (TIMS) and Parallel Accumulation Serial Fragmentation (PASEF) modes enabled. The accumulation and ramp times for TIMS were both set to 100 ms, with an ion mobility (1/k0) range from 0.6 to 1.6 Vs/cm. Spectra were recorded in the mass range from 100 to 1700 m/z. The precursor (MS) Intensity Threshold was set to 2500, and the precursor Target Intensity was set to 20,000. Each PASEF cycle consisted of one MS ramp for precursor detection followed by 10 PASEF MS/MS ramps, with a total cycle time of 1.17 s. For DIA on the timsTOF Pro, a diaPASEF scheme consisting of 34 precursor isolation windows of 26 Da width and 1 Da overlap, covering a mass range of 350–1200 m/z and an ion mobility range of 0.6 to 1.6 Vs/cm, was created using the Bruker timsControl interface (4.1).

For analysis of DDA and DIA (diaPASEF) data, the FragPipe computational proteomics platform (version 21.1) was used, with the DIA_SpecLib_Quant workflow selected [48, 49, 50, 51, 52, 53]. In this workflow, DDA data was first searched using MSFragger (version 4.1) against the Uniprot Human reference proteome reviewed entries (UP000005640; downloaded 2024_04_25) to which decoy sequences and common contaminants were appended. MSFragger features were validated with Percolator (version 3.6.4). Proteins were inferred with ProteinProphet (version 5.1.0). The spectral library created from the DDA search results with the python package EasyPQP (version 0.1.49) was used as input for DIA_NN (version 1.8.2 beta 8) for searching and quantitation of diaPASEF data. All default settings for the individual modules within this workflow were accepted. For subsequent statistical analysis, the diann output.pg_matrix file containing normalised intensities for protein groups, filtered at 1% FDR, was used as input.

### 2.6 Western Blotting

EV markers were quantified post ultracentrifugation to confirm successful EV enrichment. 20 μg of lysed precipitated EV protein was mixed with an equal volume of reducing sample buffer (125 mM Tris HCl pH 6.7, 3% SDS, 7 mM dithiothreitol, 20% glycerol, 0.05% bromophenol blue) and boiled for 5 min at 95°C, before proteins were separated on a 4%–15% polyacrylamide gradient gel and transferred onto a polyvinylidene fluoride membrane. Membranes were blocked in 5% skim milk for 2 h at room temperature, followed by 2 × 10 min washes in TBS‐T (TBS + 0.1% Tween‐20) and incubated overnight at 4°C in primary antibodies at a 1:1000 dilution (Alix‐ab186429, HSP70‐ EXOAB‐Hsp70A‐1, CD81‐ EXOAB‐CD81A‐1, CD63‐ EXOAB‐CD63A‐1, TSG101‐ ab125011, APOA1‐ sc‐376,818, Exosome Positive Control‐EXOAB‐POS‐1) and a 1:2000 dilution (Albumin‐ sc‐51,515). Monoclonal antibodies were purchased from Abcam (Cambridge, United Kingdom), System Biosciences (Palo Alto, CA, USA) and Santa Cruz (Dallas, Texas). Membranes were further washed 3 × 10 min in TBS‐T before being incubated in the dark at room temperature for 2 h with IRDye 800CW goat anti‐rabbit IgG (925–32,211, Li‐Cor Biosciences, Lincoln, NE, USA) or IRDye 680RD goat anti‐mouse IgG (925–68,070, Li‐Cor Biosciences) secondary antibodies at a 1:10,000 dilution, followed by a final 3 × 10 min TBS‐T wash and measured on the Li‐Cor Odessey CLx Imaging System (Li‐Cor Biosciences).

#### 2.6.1 O‐Link: Proximity Extension Assay—Target 48 Cytokine Panel

Plasma samples were sent to Randox, Olink commercial partner, for Proximity‐Extension‐Assay (PEA) Target 48 Cytokine Panel quantification. Olink Target 48 Cytokine Panel quantified 45 Cytokine proteins using dual recognition technology, combining antibody and DNA oligo detection/quantification methods. Absolute quantification values in pg/mL were graphed and statistical analysis completed using RStudio (version 4.2.1).

#### 2.6.2 Enzyme‐Linked Immunosorbent Assay (ELISA)

Enzyme‐Linked Immunosorbent Assays (ELISA) were used to determine soluble protein concentration in sample PPP. Sandwich ELISAs for Citrullinated Histone H3 (Clone 11D3; 501,620; Cambridge Chemicals, Cambridge, UK) and Plasminogen Activator Inhibitor 1 (Human Serpin E1/PAI‐1; DSE100; R&D Systems, Bio‐techne, Minneapolis, USA) were performed according to the manufacturer's instructions. All standards and samples were assayed in duplicate and quantified on the CLARIOstar plate reader. Data analysis was performed using the 4‐parametric logistic regression equation (4PL Curve).

### 2.7 Data and Statistical Analysis

Statistical analysis was assessed in RStudio (version 4.2.1). The EXPECT Study is a pilot study with a small sample size; therefore, normal distribution could not be assumed. A Kruskal–Wallis test was chosen to assess significance between the 3 study conditions: Baseline patients (*n* = 21), follow up DOAC (*n* = 13) and follow up LMWH treated patients (*n* = 8). Data was treated as non‐paired (default) as only a sub‐set of samples had both a baseline and follow up sample from the same patient (*n* = 14 out of 28 total enrolled). If the Kruskal–Wallis test gave significance, this value was carried forward to be corrected for multiple testing (if appropriate for assay), followed by correction for multiple comparisons between the three study conditions. If the uncorrected Kruskal–Wallis value was insignificant, it was reported without correction. A Fischer exact test was used to assess differences in categorical variables.

Statistical significance of mass spectrometry intensities was assessed using Perseus Software (version 2.0.11.0). Intensity values were log_2_ transformed and protein identifications were filtered to eliminate common contaminants, with samples grouped by their study condition (Baseline, Follow Up DOAC or Follow Up LMWH). For statistical analysis, only proteins identified in at least 50% of the replicate runs in at least one study condition were included. Differences in protein expression were determined using a *t*‐test with an FDR of 0.05 and a minimal fold change (S0) of 0.1 within the Perseus software. Data was graphed in RStudio.

## 3 Results

### 3.1 Clinical Patient Demographics

Twenty‐eight eligible patients with active cancer presenting with a VTE were recruited to the EXPECT Study, 21 of whom provided a baseline sample taken at the point of the VTE diagnosis. Follow up samples after 8 weeks on treatment were obtained from 13 patients treated with DOACs and 8 patients treated with LMWH. Fourteen patients provided paired samples of both baseline and follow up samples. The patient demographics are detailed in Table 1. The average age of the baseline cohort was 58 years (± 10.9 years SD), patients treated with DOAC was 58 years (± 9.3 years SD) and treated with LMWH was 48 years (± 14.8 years SD). The average duration of anticoagulation treatment was 8.6 (± 5.36) weeks for the DOAC group and 8.0 (± 6.05) weeks for the LMWH group. At least 50% of each cohort presented with pulmonary embolism (PE), followed by deep vein thrombosis (DVT) and other conditions. The study included a diverse range of 10 cancer types, the majority of which were metastatic. Breast cancer was the most prevalent, accounting for 33% at baseline and 46% in the DOAC follow up, but it was not observed in the LMWH group. The Khorana Score was calculated for each patient to stratify the relative risk of CAT, classifying those with < 2 points as low risk and those with ≥ 2 points as intermediate to high risk [54]. The baseline cohort showed a comparable distribution of patients with an intermediate to high‐risk Khorana score as the cohort of patients receiving DOACs (48% vs. 31%). However, unavoidably, the proportion of patients receiving LMWH with an intermediate to high‐risk score was slightly higher, at 62.5%.

**TABLE 1:** Patient demographics of enrolled participants in the EXPECT study at baseline, treated with DOACs compared to patients treated with LMWH.

Characteristics (mean ± SD)	Baseline (*n* = 21)	Follow Up DOAC (*n* = 13)	Follow Up LMWH (*n* = 8)
Age (years)	58.0 ± 10.91	58.2 ± 9.32	48.2 ± 14.89
Gender, *n* (%)			
Male	5 (24%)	2 (15%)	3 (38%)
Female	16 (76%)	11 (85%)	5 (62%)
Thrombosis type, *n* (%)			
DVT	5 (23%)	4 (31%)	1 (12.5%)
PE	13 (62%)	9 (69%)	4 (50%)
DVT and PE	2 (10%)	0	3 (37.5%)
Other	1 (5%)	0	0 (%)
Time on treatment (weeks)	N/A	8.6 ± 5.36	8.0 ± 6.05
BMI (kg/m^2^)	28.8 ± 6.29	28.7 ± 7.84	28.0 ± 6.53
Smoking, *n* (%)			
Current smoker	1 (5%)	1 (8%)	0 (0%)
Ex‐smoker	4 (19%)	2 (15%)	2 (25%)
Non‐smoker	9 (43%)	3 (23%)	2 (25%)
Data unknown	7 (33%)	7 (54%)	4 (50%)
Aspirin use, *n* (%)			
Yes	1^a^ (5%)	0 (0%)	0 (0%)
No	20 (95%)	13 (100%)	8 (100%)
Cancer type, *n* (%)			
Breast	7 (33%)	6 (46%)	0
Colorectal	3 (14%)	2 (15%)	1 (12.5%)
Ovarian	3 (14%)	1 (8%)	2 (25%)
Endometrial	2 (10%)	1 (8%)	1 (12.5%)
Prostate	1 (5%)	1 (8%)	0
Lung	2 (10%)	0	1 (12.5%)
NSC lung	1 (5%)	1 (8%)	1 (12.5%)
Melanoma	0	0	1 (12.5%)
Testicular	0	0	1 (12.5%)
Non‐Hodgkins lymphoma	1 (5%)	0	0
Pseudomyxoma peritonei (PMP)	1 (5%)	0	0
Colorectal + lung	0	1 (8%)	0
Cancer stage, *n* (%)			
Metastatic	16 (76%)	9 (69%)	8 (100%)
Stage II	4 (19%)	3 (23%)	0
Stage III	1 (5%)	1 (8%)	0
Major Bleeding in the past month, *n* (%)			
Yes	0	0	0
No	21 (100%)	13 (100%)	8 (100%)
Bleeding at diagnosis of CAT, *n* (%)			
Yes	0	0	0
No	21 (100%)	13 (100%)	8 (100%)
Surgical intervention in the last 2 months, *n* (%)			
Yes	7 (33%)	3 (23%)	3 (37.5%)
No	14 (67%)	10 (77%)	5 (62.5%)
Immobility > 4 days (non‐surgical), *n* (%)			
Yes	2 (10%)	0 (%)	1 (12.5%)
No	17 (80%)	12 (92%)	6 (75%)
Data unknown	2 (10%)	1 (8%)	1 (12.5%)
History of VTE, *n* (%)			
Yes	2 (10%)	1 (8%)	2 (25%)
No	15 (71%)	9 (69%)	3 (37.5%)
Data unknown	4 (19%)	3 (24%)	3 (37.5%)
Khorana Score, *n* (%)			
< 2; low risk	11 (52%)	9 (69%)	3 (37.5%)
≥ 2; intermediate to high risk	10 (48%)	4 (31%)	5 (62.5%)

Abbreviations: BMI, body mass index; CAT, cancer associated thrombosis; SD, standard deviation; VTE, venous thromboembolism.

^a^ Aspirin dose 75 mg per, < 100 mg therefore eligible for inclusion.

### 3.2 Circulating EV Size and Concentration Remain Relatively Constant Between DOAC and LMWH Treatment in CAT Patients

**FIGURE 2:**
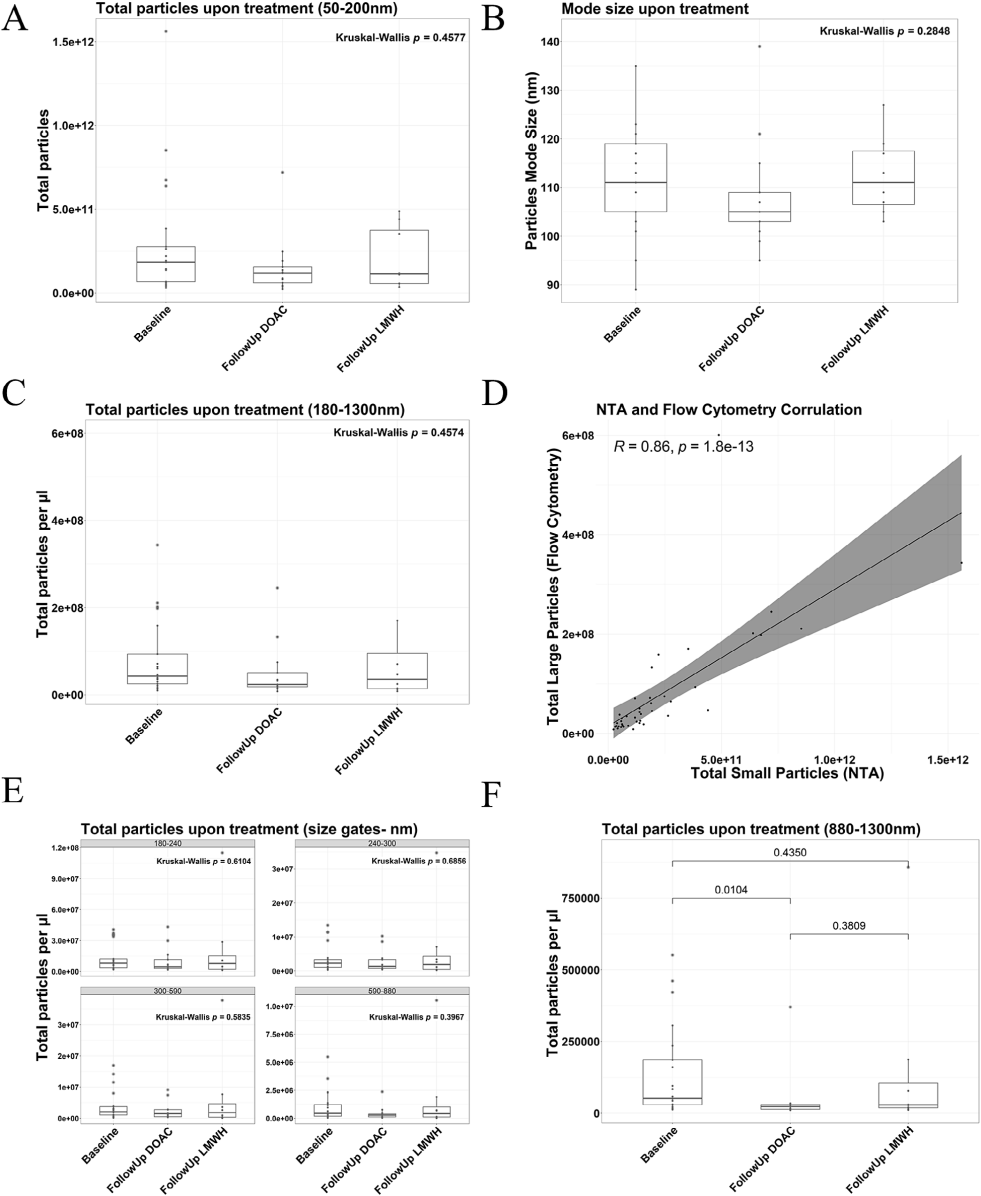
CAT patients treated with DOACs exhibit a similar size and concentration of circulating EVs when compared to patients treated with LMWH. Vesicle quantification and sizing of circulating small EVs (50–200 nm) in PPP was assessed using Nanoparticle Tracking Analysis (NTA) with the Nanosight NS300 (Baseline *n* = 21; Follow Up DOAC *n* = 13; Follow Up LMWH *n* = 8). 15 × 60 s videos were captured at a camera level of 13 and analyzed using a detection threshold of 10. (A) Small EV concentration (Kruskal–Wallis test, *p* = 0.4577) and (B) size (Kruskal–Wallis test, *p* = 0.2848) remained constant between baseline and follow up samples from cohorts treated with both DOAC and LMWH. (C) Flow Cytometry was performed with the CytoFlex S to quantify large EVs across 180–1300 nm size in PPP (Baseline *n* = 21; Follow Up DOAC *n* = 13; Follow Up LMWH *n* = 8; Kruskal–Wallis test, *p* = 0.4574). Light scatter intensities were adjusted to reflect biological EV properties using Rosetta calibration beads and software. (D) A strong statistically significant correlation was observed between the NTA and total Flow Cytometry EV counts (*R* = 0.86; *p* = 1.8e‐13). (E, F) Size gates were constructed using polystyrene beads to quantify EVs between 180–240 nm, 240–300 nm, 300–590 nm, 590–880 nm, and 880–1300 nm, with statistically significantly lower EVs sized between 880–1300 nm in the DOAC arm when compared to baseline (880–1300 nm EV size gate only, Kruskal–Wallis test, *p* = 0.0257).

The concentration of small and large EVs was quantified in the baseline and follow‐up groups to determine if either DOAC or LMWH treatment attenuated global circulating EV profiles. There was no significant deviation in the concentration of small EVs between groups quantified via Nanoparticle Tracking Analysis, which was followed by the quantification of the large EVs by flow cytometry (Figure 2A,C). The small and large EV quantification showed a statistically significant strong positive correlation (Figure 2D: *r* = 0.86; *p* = 1.8 × 10^−13^). A slight trend in lower circulating EVs in the DOAC arm was observed in both the small and large EVs; however, significance was not reached (Figure 2A: small EVs; *p* = 0.4577, Figure 2C: large EVs; *p* = 0.4574). The small EV mode size was numerically lower in the DOAC arm compared to LMWH (Figure 2B: *p* = 0.2848). Interestingly, upon quantification of large EVs separated into incremental size gates (Figure 2E), the largest EV size bin of 880–1300 nm gave significantly lower EVs in the DOAC arm compared to LMWH (Figure 2F: *p* = 0.0257). Taken together, circulating EV size and concentration remain relatively constant between DOAC and LMWH treatment; however, there was a noticeable trend in lower EV concentration and size in the DOAC arm.

### 3.3 Endothelial‐Derived Large EVs Remain Stable Upon DOAC Treatment to the Same Degree as LMWH, With a Potential Trend Towards a DOAC‐Mediated Reduction of Tissue Factor Expressing EVs

**FIGURE 3:**
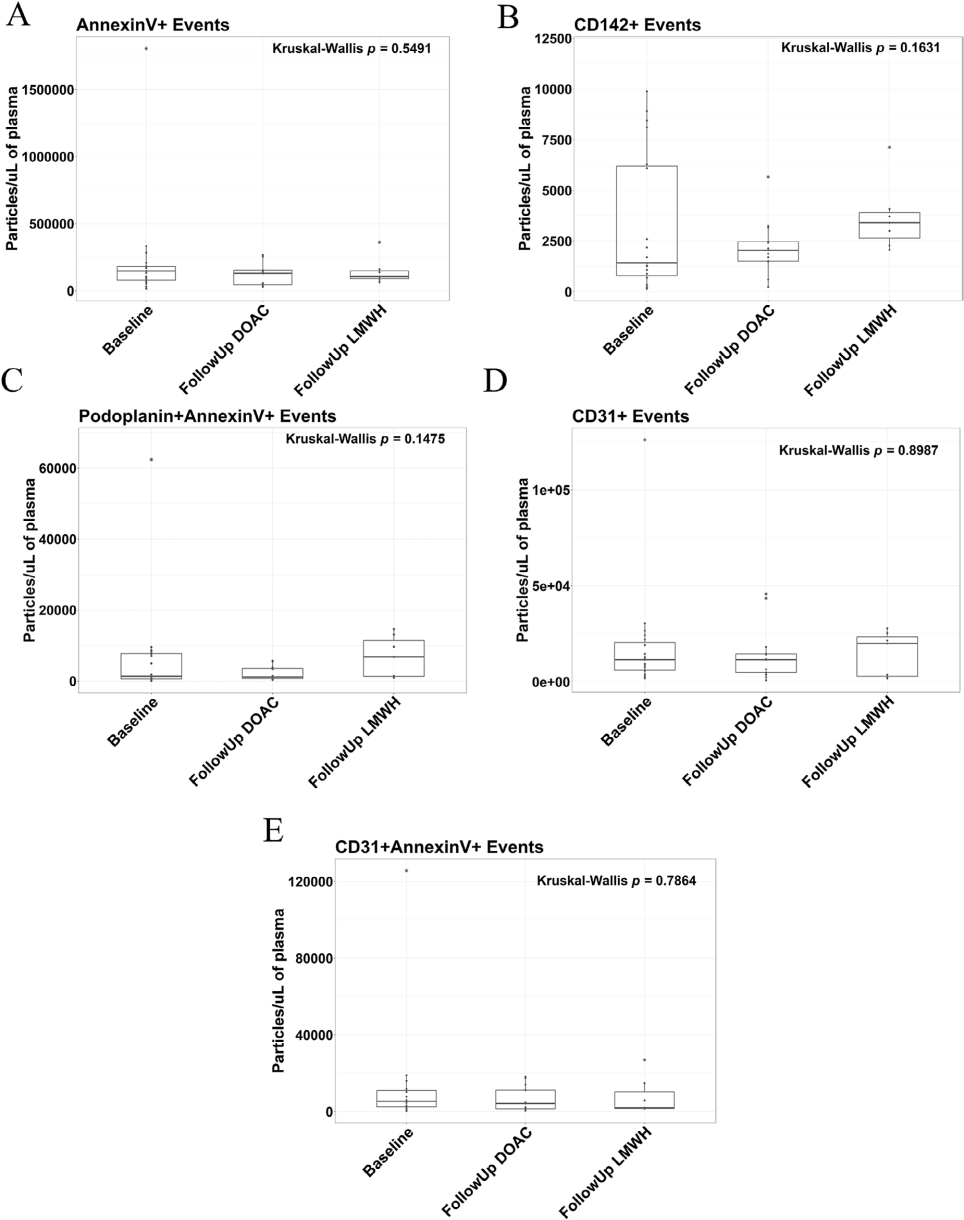
Endothelial‐derived large EVs remain stable in CAT patients upon treatment with DOAC and LMWH. Endothelial markers of CD142, Podoplanin, and CD31, along with Annexin V, were used to quantify large endothelial‐derived EVs by Flow Cytometry in CAT patients treated with DOACs compared to LMWH (Baseline *n* = 19; Follow Up DOAC *n* = 13; Follow Up LMWH *n* = 7). EVs were crudely isolated from PPP via 20,800 × *g* benchtop centrifugation and washed with PBS before being stained with fluorescently‐bound antibodies in Annexin V Binding Buffer and quantified on the CytoFlex S. (A) Phosphatidylserine‐expressing EVs (AnnexinV) remained constant between anticoagulant treatments. (B) CD142 expression did not significantly change; however, there was a slight reduction in the DOAC arm compared to LMWH treatment. (C) Podoplanin co‐expressed with Annexin V expression followed a similar trend. (D, E) CD31 expression and co‐expression with Annexin V remained constant between the anticoagulant treatment arms.

To investigate the levels of endothelial as well as platelet‐derived EVs under such treatment conditions, flow cytometry immunofluorescent staining was carried out. It is known that tissue factor (CD142) and podoplanin‐expressing EVs contribute to cancer‐associated thrombosis [21]; however, a comparison of the gold standard anticoagulant effect of such mechanisms is unknown. We show a statistically insignificant but numerical trend in a DOAC‐mediated reduction in tissue factor‐expressing large EVs compared to the LMWH treatment group (Figure 3B: *p* = 0.1631); however, podoplanin co‐expressed with AnnexinV remains stable (Figure 3C: *p* = 0.1475). CD31 (PECAM) and CD31+/AnnexinV+ EVs derived from endothelial cells remained remarkably stable between groups (Figure 3D,E: *p* = 0.8987; *p* = 0.7864), along with AnnexinV expressing EVs (Figure 3A: *p* = 0.5491). We conclude that endothelial‐derived EVs are unaffected by DOAC or LMWH anticoagulant treatment; however, there is a potential that tissue factor‐expressing EVs are attenuated by DOAC treatment, with further studies needed to confirm such observed trends.

### 3.4 Prothrombotic EVs Are not Altered by DOAC or LMWH Treatment, of Which the Majority Are Potentially Megakaryocyte‐Derived

**FIGURE 4:**
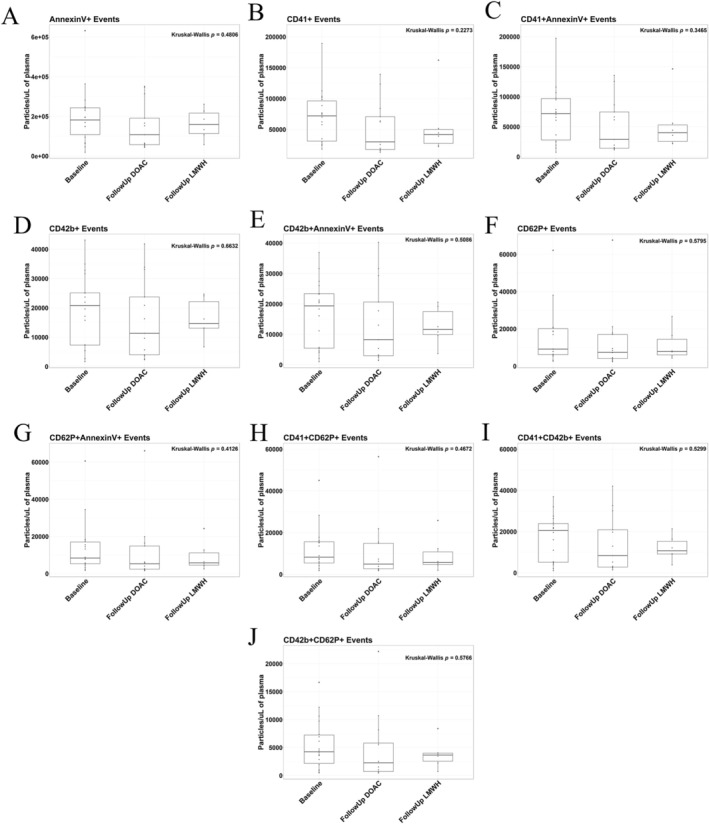
Platelet‐derived large EVs remained stable in CAT patients upon treatment with both DOAC and LMWH anticoagulation. Platelet markers of CD41, CD42b, and CD62P, along with Annexin V, were used to quantify large platelet‐derived EVs by Flow Cytometry in CAT patients treated with DOACs compared to LMWH (Baseline *n* = 18; Follow Up DOAC *n* = 13; Follow Up LMWH *n* = 6). EVs were crudely isolated from PPP via 20,800 × *g* benchtop centrifugation and washed with PBS before being stained with fluorescently bound antibodies in Annexin V Binding Buffer and quantified on the CytoFlex S. (A–G) Phosphatidylserine‐expressing EVs (AnnexinV), along with EVs expressing CD41, CD42b, and the platelet activation marker of CD62P, remained constant between anticoagulant treatments. (H–J) Dual expression of platelet markers was also found to remain constant between drug groups.

**FIGURE 5:**
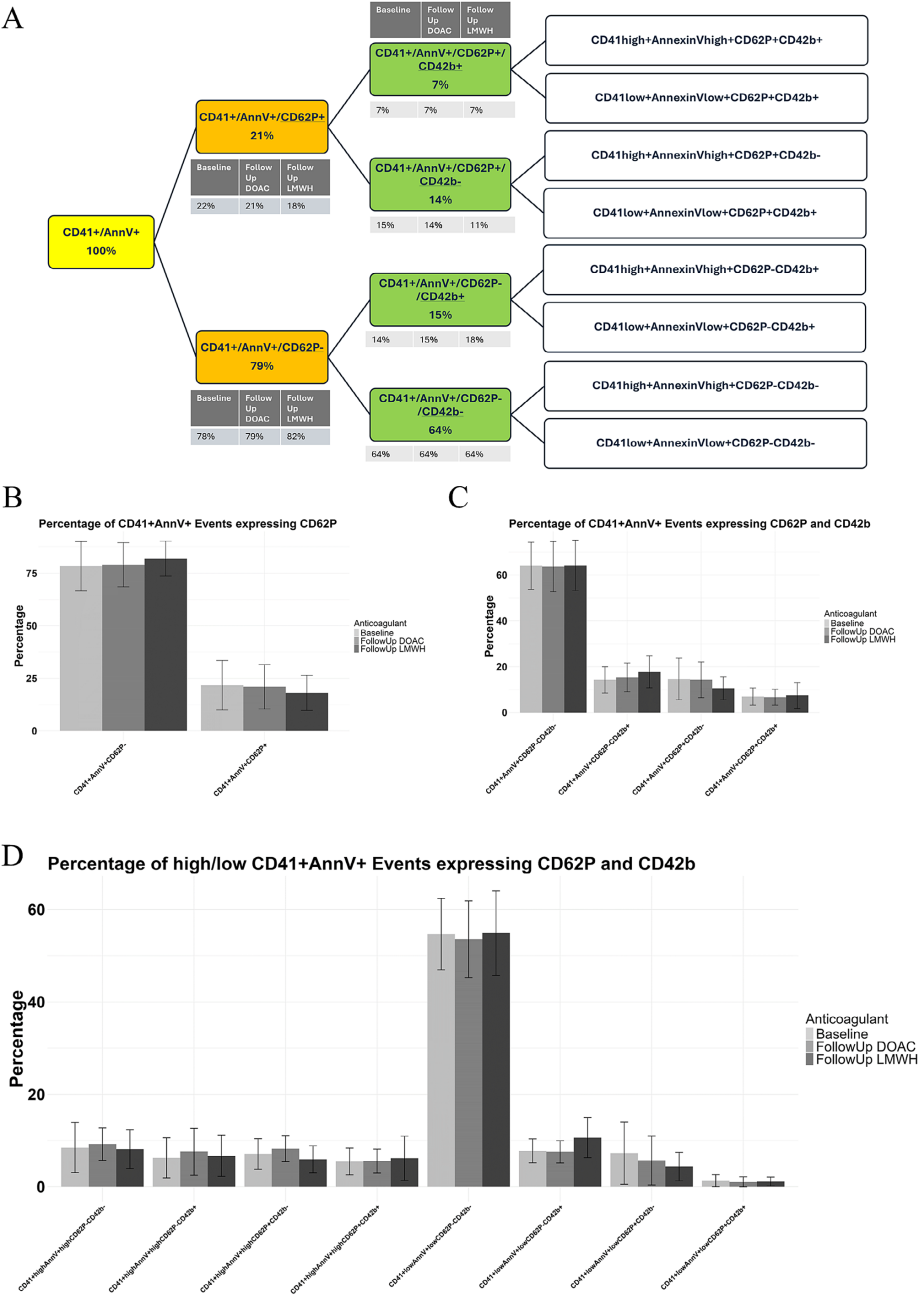
The platelet‐derived EV environment in anticoagulated cancer‐associated thrombosis patients reveals that the majority of such EVs are megakaryocyte‐derived. Platelet markers of CD41, CD42b, and CD62P, along with Annexin V, were used to quantify large platelet‐derived EVs by Flow Cytometry in CAT patients treated with DOACs compared to LMWH (Baseline *n* = 18; Follow Up DOAC *n* = 13; Follow Up LMWH *n* = 6). EVs were crudely isolated from PPP via 20,800 × *g* benchtop centrifugation and washed with PBS before being stained with fluorescently‐bound antibodies in Annexin V Binding Buffer and quantified on the CytoFlex S. (A) The flow diagram outlines the breakdown of total CD41 + Annexin V+ EVs, representing the total of quantified platelet‐derived EVs for this analysis. (B) Proportion of the CD41 + Annexin V+ total EVs that express CD62P. (C) Proportion of the CD41 + Annexin V+ total EVs that express CD62P and CD42b. (D) Proportion of high and low CD41 + Annexin V+ expression for CD62P and CD42b events.

We found that platelet‐derived EVs remain consistently stable with no observed deviations under either anticoagulant treatment (Figure 4A–J). Therefore, we aimed to characterise and classify platelet‐derived EVs within this stable CAT cohort through characterising the EV landscape (Figure 5A–D). Taking CD41 + AnnexinV+ platelet EVs as 100%, we showed 79% of such EVs are negative for CD62P, which are proposed to be megakaryocyte‐derived [55, 56](Figure 5A,B). Furthermore, 64% are negative for both CD62P and CD42b, highlighting a large megakaryocyte‐derived EV population within this cohort (Figure 5A,C), which is echoed in Figure 5D, showing low CD41/AnnexinV expression. Literature reveals megakaryocyte EVs are capable of infiltrating the bone marrow as well as other bodily systems including the spleen and liver, shown to interact with haematopoietic stem and progenitor cells enhancing cellular processes including platelet biogenesis [57].

### 3.5 The CAT EV Cargo Proteome Remains Remarkably Stable Under Both DOAC and LMWH Anticoagulant Treatment

**FIGURE 6:**
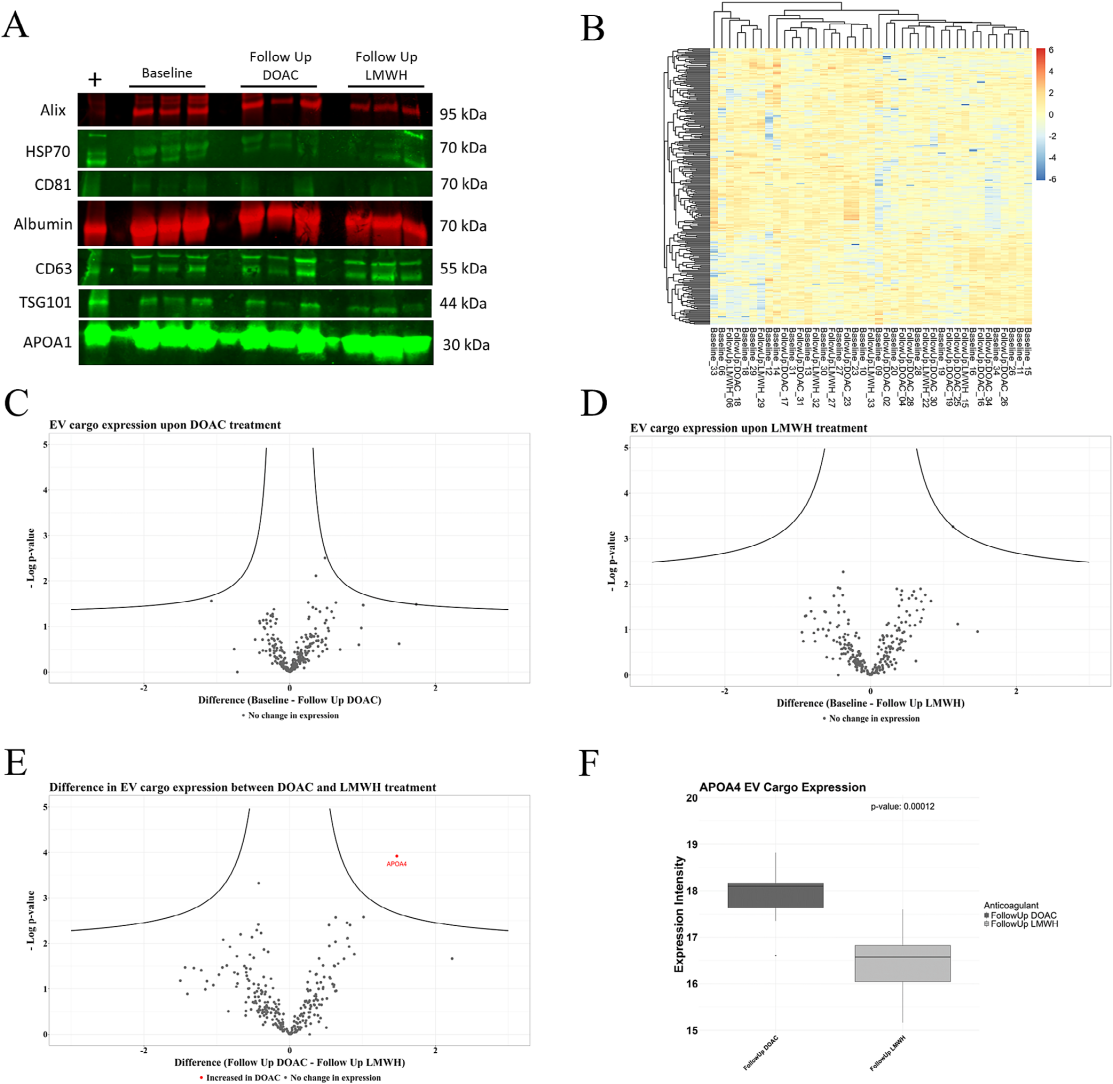
EV cargo proteomic expression levels remain stable in CAT patients upon anticoagulation with both DOAC and LMWH treatment. EVs were enriched from PPP via ultracentrifugation (6 h at 52,000 rpm at 4°C–corresponding to ~120,000 × *g*) followed by mass spectrometry to quantify cargo proteome and western blot validation (Baseline *n* = 21; Follow Up DOAC *n* = 13; Follow Up LMWH *n* = 7). (A) Enriched EVs were lysed, 20 μg of protein loaded onto a 4%–15% gradient SDS‐polyacrylamide gel, immunoblotted for transmembrane and soluble vesicle markers confirmed the presence of EVs in the enrichment (Alix–1:1000, HSP70–1:1000, CD81–1:1000, Albumin–1:2000, CD63–1:1000, TSG101–1:1000, APOA1–1:1000). Data independent acquisition (DIA) revealed stable protein expression within the EV cargo under both DOAC and LMWH treatment, with 257 proteins identified that were present in at least 50% of at least one treatment group. (B) A heatmap of all cargo proteins shows no clustering between treatment arms, highlighting the stable and unchanged EV cargo upon short term anticoagulation. (C–E) Volcano plots visualised the differential expression of proteins between treatments, showing significant expression above the black curve (threshold for statistical significance was set using an FDR of 0.05 and S0 of 0.1). There were no differentially expressed proteins upon DOAC or LMWH treatment between baseline and follow up samples, however, APOA4 was significantly increased in the DOAC treated patient EVs compared to LMWH treatment at follow up (F, *p* = 0.00012).

We then carried out proteomic analysis of the EV cargo proteins between conditions and found a remarkably stable proteome under both DOAC and LMWH treatment, with 257 proteins identified that were present in at least 50% of at least one condition (Figure 6B–F). We showed successful EV enrichment in all conditions through immunoblotting for EV markers of Alix, HSP70, CD81, CD63, and TSG101 (Figure 6A). A heatmap of these 257 proteins showed no clustering between the 3 groups, highlighting a stable CAT proteome (Figure 6B). Volcano plots of the −log(*p*) showed no differentially expressed proteins upon DOAC or LMWH treatment compared to baseline, further highlighting this stable proteome (Figure 6C–E), APOA4, known for its roles in lipid metabolism and anti‐inflammatory properties [58, 59], was significantly increased in the DOAC treated patient EVs compared to LMWH treatment (Figure 6F).

The CAT inflammatory profile is comparable between DOAC and LWMH treatment with a numeric trend in lower cytokine expression in the DOAC arm, which is associated with a reduction and moderate correlation with circulating citrullinated Histone H3 levels.

**FIGURE 7:**
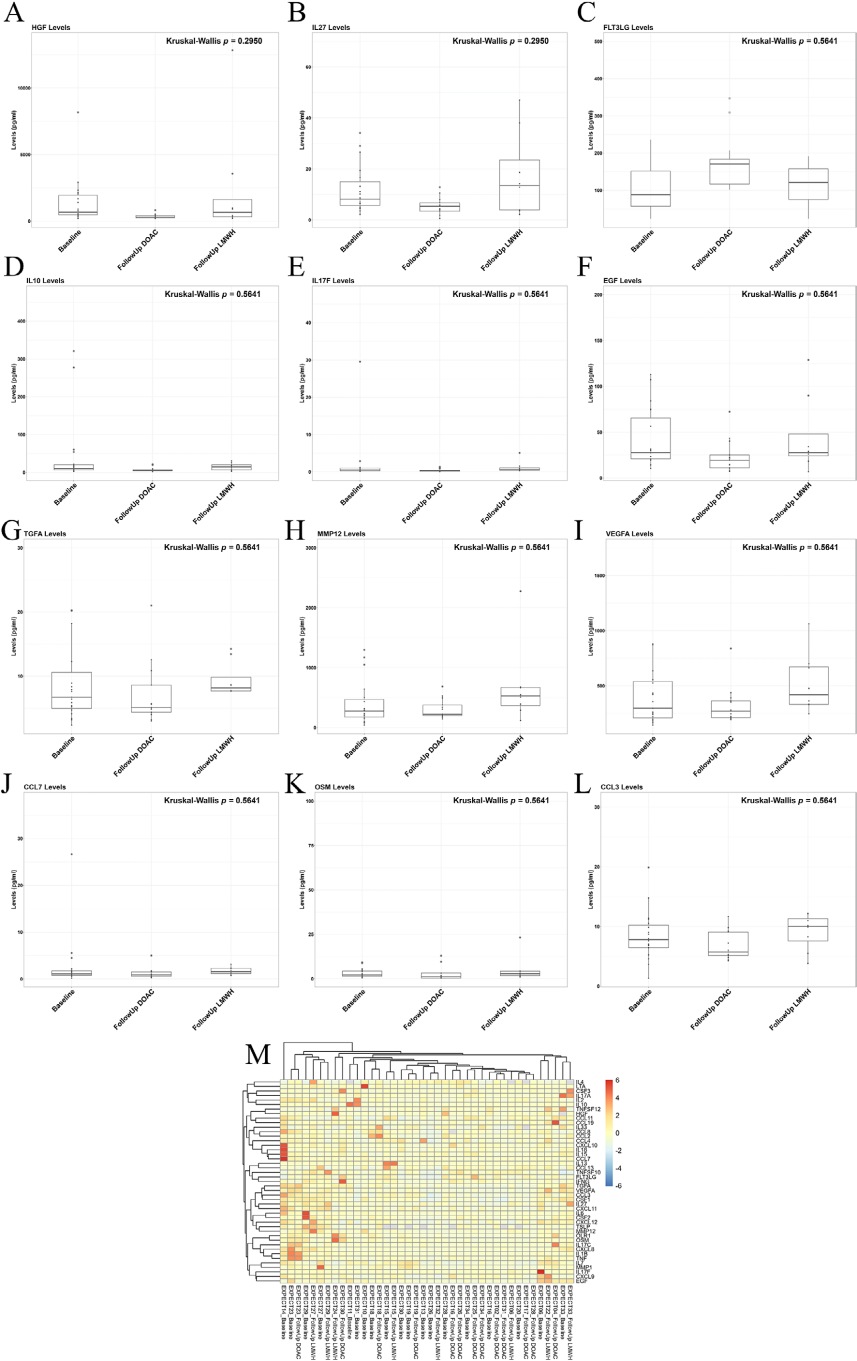
Investigating the circulating cytokine profile of CAT patients treated with DOACs compared to LMWH, with a potential trend in a DOAC‐mediated reduction. (A–L) Olink Proximity Extension Assay (PEA) quantified the absolute value of a panel of 45 inflammatory proteins on the T48 Cytokine Panel at baseline and post‐treatment with either DOACs or LMWH (Baseline *n* = 19; Follow Up DOAC *n* = 13; Follow Up LMWH *n* = 8). Statistics of all proteins are found on Table S1 with the top 12 hitting proteins visualised via boxplots. (M) Heatmap of inflammatory profiles showing comparable expression between treatment arms.

Echoing results from the EV landscape, a statistically insignificant but numeric trend towards lower cytokine levels was observed in plasma of patients treated with DOAC compared to LMWH (Figure 7A–M; Table S1). This numeric reduction was found in 30 of the 45 cytokines quantified in the DOAC arm compared to both baseline and LMWH treatment, of which importantly included HGF, IL27, IL17F, EGF, TGFA, MMP12, VEGFA, CCL7, CCL8, IL6, and IL18 (Table S1). Results are intriguing and align with the previously reported anti‐inflammatory properties of DOAC [41, 43]; however, 8 weeks on treatment might not be sufficient time to elicit a significant response. Moreover, Hepatocyte Growth Factor (HGF) levels exhibited the largest reduction in the DOAC compared to LMWH treatment of all the cytokines quantified, approaching significance, and demonstrated a moderate and statistically significant correlation with plasma levels of citrullinated Histone H3 (CitH3, Figure S1C). CitH3 levels echoed the overall inflammatory and HGF profile, significantly dropping upon DOAC treatment compared to both baseline and LMWH cohorts; however, CitH3 in the LMWH arm did not deviate from baseline levels (Figure S1A: *p* = 0.0092). High CitH3 levels have previously been associated with poor prognosis as well as neutrophil extracellular trap release, which is known to promote thrombotic events [60, 61, 62], and results could point to involvement of such mechanisms. This is an intriguing result, which needs to be validated in a larger CAT population.

## 4 Discussion

To our knowledge, this is the first study to compare the EV and inflammatory profiles of DOAC compared to LMWH treatment under CAT conditions. We have shown that treatment with either anticoagulant maintained a similar hypercoagulable and prothrombotic EV and cytokine profile. We did observe a trend suggesting DOAC‐mediated reductions, which would merit further future investigation in larger studies. This pilot observational study has sparked hypothesis‐generating research into the mechanistic pathways underlying the clinical findings of reduced VTE recurrence with DOACs, as reported in the Hokusai, SELECT‐D, and Caravaggio trials [8, 9, 10]. Our results highlighted a significant reduction in large EVs (880–1300 nm) with DOAC treatment, alongside numerical trends in total EVs and tissue factor‐expressing EV levels. Characterisation of the platelet EV landscape suggested the majority were megakaryocyte‐derived, with a stable EV cargo proteome. Additionally, while inflammatory profiles remained steady, there was a trend towards reduced inflammatory cytokines with DOACs compared to LMWH. This mechanistic data falls in line with the clinical data which highlighted treatment with DOACs as noninferior to LMWH at preventing recurrent VTE in the cancer setting. This study supports this shift in the anticoagulant treatment choice from LMWH to DOAC in the cancer setting.

There is very little evidence as to the anticoagulant effect on circulating EVs, especially in the cancer‐associated VTE setting. Considering the potent role they play in thrombotic and cancer mechanisms, we deemed them an essential area to investigate. The randomised phase II Microtec Study evaluated the cumulative incidence of VTE in advanced cancer with lower levels of tissue factor EVs not on thromboprophylaxis enoxaparin (LMWH) compared to patients with higher tissue factor EVs (TF‐EVs) randomised to enoxaparin or observation [63]. They showed the higher TF‐EV cohort on enoxaparin had a VTE cumulative incidence at 8 weeks of 5.6%, whereas the high TF‐EV observational cohort gave a cumulative incidence of 27.2% (*p* = 0.06). Enoxaparin treatment seemed to bring down the VTE incidence to the same level as the low TF‐EV cohort, which gave a cumulative VTE incidence of 7.2%. These results show cancer patients with higher TF‐EVs were 7 times more likely to develop a VTE in the observational cohort than those with the same TF‐EV concentration who received enoxaparin, highlighting that LMWH might be causing a reduction of TF‐EVs [63]. Similar work by Remedios et al. also showed a significant reduction of TF‐EVs upon 3‐month treatment for cancer‐associated DVT with LMWH compared to a vitamin K antagonist [64]. Interestingly, DOAC treatment has also been shown to be associated with a reduction of circulating EVs, investigated with rivaroxaban and apixaban [41], but our work being the first to investigate such under DOACs as a whole drug group compared with LMWH or in the cancer‐associated VTE setting. Previous work from our lab by Weiss et al. has shown rivaroxaban anticoagulation in non‐valvular atrial fibrillation patients reduced circulating EVs compared to warfarin treatment, which showed an anti‐inflammatory phenotype [41]. Furthermore, Featherby et al. showed incubation of apixaban, but interestingly not rivaroxaban, with two cell lines decreased the release of tissue factor expressing EVs. Moreover, this co‐incubation of apixaban with both cell lines reduced the rate of proliferation and resulted in a complete inhibition of FVIIa proteolytic activity (however, this was not observed with rivaroxaban). This direct inhibition of TF‐FVIIa activity resulted in reduced cancer cell proliferation, indicating potential anti‐tumoral effects of apixaban [65]. Taking this with our results showing a significant reduction in the largest EV size gate (880‐1300 nm) upon DOAC treatment compared to baseline, which was not caused by platelet or endothelial derived EVs, potentially highlights the anti‐tumour effects of DOAC treatment reducing these large potentially tumour derived EVs in circulation. These studies show both DOAC and LMWH are associated with a reduction of circulating and specifically tissue‐factor expressing EVs in the cancer setting, with our results showing a numeric trend in a DOAC‐mediated reduction, which must be validated with larger studies.

Upon evaluation of this CAT platelet derived EV environment, the majority (79%) of EVs expressing the platelet marker CD41 and Annexin V were negative for CD62P (P‐selectin). It is proposed that these EVs are megakaryocyte derived, as it has been previously demonstrated that platelets release EVs only upon activation; however, megakaryocytes release EVs expressing CD41 + Annexin V + CD62P‐into circulation consistently [55]. Flaumenhaft et al. elegantly showed that megakaryocytes release CD41 + Annexin V + CD62P‐EVs consistently, whereas platelets require stimulation to undergo EV biogenesis [56]. It was shown that the treatment of megakaryocytes with the microtubule inhibitor nocodazole, known to inhibit microtubule assembly and attenuate platelet production, does not affect megakaryocyte EV release, promoting the hypothesis that these are two distinct processes [56]. It is known that platelet‐derived EVs can infiltrate the bone marrow under inflammatory conditions, become endocytosed by megakaryocytes, and alter megakaryopoiesis [66]; however, the opposite has also been observed. IV injection of megakaryocyte EVs into mice triggered platelet biogenesis, showing significantly increased platelet levels 16 h after administration [57]. Authors also importantly highlight that megakaryocytes are found in distinct areas of the body outside the bone marrow, including the lungs, spleen, and liver [57]. These studies reveal the diverse nature of megakaryocyte EVs, with the healthy profile as well as the extent to which they are affected by pathological states remaining unclear, all of which needs to be investigated with future studies.

The role of inflammation in both malignancy and thrombosis is well established, enhancing the progression of both pathophysiological states [33, 67], encapsulating an essential mechanism to study in the treatment of cancer‐associated thrombosis. Platelets and their EVs express and carry a multitude of pro‐and anti‐inflammatory cytokines, which upon release initiate an immune response, promote further platelet reactivity, and activate the coagulation cascade, all of which initiate positive feedback loops [67, 68, 69, 70, 71, 72]. Immune activation and inflammation are necessary to target malignant cells; however, under chronic persistent dysregulation, tumour cells grow, proliferate, and migrate [73, 74]. Anti‐inflammatory properties, altering the levels of these cytokines, have the potential to curb recurrent thrombosis by suppressing these pathways as well as potentially suppressing EV release, tumour growth, and metastasis [67, 75, 76, 77].

Some evidence has shown anti‐inflammatory effects of both DOAC and LMWH treatment, with our work showing these anticoagulant drugs do not significantly affect cytokine levels; however, the potential intriguing DOAC‐mediated reduction needs to be validated in larger CAT cohorts. Furthermore, we showed APOA4 was significantly elevated in the DOAC treated patient EV cargo compared to LMWH, which has been shown to have anti‐inflammatory effects [58, 59]. Inflammatory profiles of DOACs have mainly been studied in rivaroxaban [78]. Plasma levels of inflammatory cytokines of IL‐2, IL‐4, IL‐10, TNF, and IFN‐γ were reduced upon rivaroxaban treatment compared to warfarin in atrial fibrillation patients, with levels still slightly higher than controls [78]. Similar results were shown in the X‐VeRT Study with a rivaroxaban mediated reduction in IL6 and CRP in nonvalvular atrial fibrillation patients [79]. Furthermore, mice and cell culture studies, including vascular smooth muscle cells, have shown decreased mRNA expression of inflammatory cytokines such as IL‐6, MCP‐1, IL‐1β, and TNF‐α in samples exposed to rivaroxaban compared to control samples [80, 81, 82]. Few studies investigating the inflammatory profiles of the other DOACs showed similar results [67, 83, 84]. Furthermore, LMWH has been shown to exert similar anti‐inflammatory as well as anti‐tumorigenic and metastatic pleiotropic effects [42, 85, 86, 87]. Along with the ability to stimulate the tissue‐factor inhibitory pathway (TFPI) from endothelial cells suppressing thrombosis, it can also bind to cytokines, preventing interactions with receptors and downregulating inflammation [42, 88]. This highlights its role not only in haemostasis but also in immune modulation, which may have implications in pathological conditions such as cancer‐associated thrombosis [42, 88]. Of such, LMWH binds to and blocks P‐selectin, inhibiting leukocyte activation and adhesion to the endothelium, an important process for tumour evasion and thrombosis, subsequently inhibiting immune mediated inflammation [89, 90, 91]. Hochart et al. showed a reduction in levels of proinflammatory cytokines of TNF‐α, IL‐8, IL‐6, and IL‐1β as well as NF‐κB translocation upon LMWH treatment to cultured monocytes after stimulation by lipopolysaccharide [85]. The use of LMWH for its anti‐inflammatory purposes was highlighted during the COVID‐19 pandemic to combat the viral cytokine storm [92, 93, 94]. Of prominent importance, LMWH binds to and decreases IL‐6, the key cytokine in COVID19 severity, highlighting its ability to reduce overall inflammation [92]. Taking these results, along with the numeric trend in a DOAC‐mediated cytokine reduction we have shown, it still remains to be confirmed if DOACs and LMWH have similar pleiotropic anti‐inflammatory properties in this patient cohort.

There are understood limitations to the EXPECT Study, which are important to note. First, the observational nature of this study, with anticoagulant choice determined by the physician's clinical judgement, naturally led to a separation of patient cohorts. LMWH is typically prescribed to patients with higher bleeding risks, gastrointestinal malignancies, kidney or liver dysfunction, thrombocytopenia, or drug–drug interactions [95], resulting in a higher proportion of LMWH patients with intermediate to high‐risk Khorana scores (≥ 2). Second, the pilot nature of the study means it is underpowered, making it challenging to reach a significant threshold of 5%; therefore, *p*‐values should be interpreted irrespective of such [96]. This study comprises a wide variation of important clinical factors, including cancer types (ranging from low thrombotic risk of breast cancer to the moderate to high thrombotic risk of lung cancer [97]), treatment options, treatment success, and comorbidities (data unknown), which increased the spread of our data, likely masking observable effects. Additionally, the persistent malignancy and metastatic nature of many patients also contribute to sustained cancer‐derived EV release and inflammation, which may have further diminished detectable changes within the 8‐week study window. Our findings suggest that 8 weeks of anticoagulation fails to counteract the severe hypercoagulable state observed, likely reflecting the advanced, chronic nature of these patients. Results from our lab have previously shown a marked but statistically insignificant negative correlation between circulating platelet markers (soluble CD62P, TSP‐1, and PF4) and time on rivaroxaban treatment (10–50 months) in patients with nonvalvular atrial fibrillation (Figure S1) [41]. These findings suggest that DOAC‐mediated protective effects may become more evident over time, indicating that 8 weeks of anticoagulation in the present study may have been insufficient to observe measurable effects. This aligns with our observation that patients with severe hypercoagulability had unchanged EV and cytokine profiles, possibly reflecting extreme vascular dysregulation. Lastly, this study focused on EV and cytokine profiles as mechanisms driving hypercoagulability and VTE development, but additional pathways—such as NETosis and thrombin generation potential—may also contribute to the reduction in VTE incidence under DOAC treatment observed in clinical trials. These limitations, though unavoidable due to the observational and pilot nature of the study, are essential to emphasise.

To conclude, DOAC treatment is associated with pleiotropic effects on hypercoagulable and prothrombotic mechanisms, similar in extent to those observed with LMWH treatment. In clinical practice, the shift from LMWH to DOACs has been endorsed due to the easier route of administration and hence compliance of patients, supporting this clinical shift in treatment choice in the cancer‐associated thrombosis setting.

## Author Contributions

All authors contributed to the study as follows: conceptualization (B. Kevane, F. Ní Áinle, P.B. Maguire), methodology (H. Macleod, N. Copty, D. Doherty, L. Weiss, E. Fouhy, R. Power, N. Ryan, K. Saeed, E. O'Rourke, R. Faryal, S. Kelliher, B. Kevane, F. Ní Áinle, P.B. Maguire), software (H. Macleod, L. Weiss, E. Fouhy, S. Kelliher), data curation (H. Macleod, N. Copty, D. Doherty, R. Power, N. Ryan, K. Saeed, R. Faryal, S. Kelliher), investigation (B. Kevane, F. Ní Áinle, P.B. Maguire), validation (H. Macleod, L. Weiss, S. Kelliher), formal analysis (H. Macleod, L. Weiss, E. Fouhy, S. Kelliher), supervision (L. Weiss, B. Kevane, F. Ní Áinle, P.B. Maguire), funding acquisition (F. Ní Áinle, P.B. Maguire), visualisation (H. Macleod), project administration (H. Macleod, N. Copty, D. Doherty, N. Ryan, K. Saeed, E. O'Rourke, R. Faryal, F. Ní Áinle, P.B. Maguire), resources (N. Copty, R. Power, E. O'Rourke, S. Kelliher, B. Kevane, F. Ní Áinle, P.B. Maguire), writing – original draft (H. Macleod), and writing – review and editing (H. Macleod, F. Ní Áinle, P.B. Maguire).

## Ethics Statement

Patients were recruited to the EXPECT Study following ethical approval granted by the Research and Ethics Committee Board in the Mater Misericordiae University Hospital (MMUH: Ref 1/378/2169: 21/03/2022), with an approved amendment to add an additional timepoint sample on 16/02/2023. Informed written consent according to the Declaration of Helsinki was obtained prior to sample acquisition.

## Conflicts of Interest

This work was funded by a research grant from Daiichi Sankyo to F. Ní Áinle and P.B. Maguire.

## Supporting information


**Figure S1.** Citrullinated histone H3 and PAI levels in CAT patients treated with DOACs compared to LMWH. Immunoassays quantified hypercoagulable markers of citrullinated histone H3 (CitH3) and PAI‐1 in PPP of CAT patients treated with DOAC compared to LMWH treatment. (A) Results show a significant reduction in CitH3 in the DOAC arm compared to both baseline and LMWH, however levels in the LMWH arm did not deviate from baseline (Kruskal–Wallis test, *p* = 0.009242). (B) PAI‐1 remained constant between both anticoagulant drugs (Kruskal–Wallis test, *p* = 0.3226). (C) A statistically significant moderate correlation was observed between CitH3 and the inflammatory protein HGF from the Olink analysis (*R* = 0.54, Spearman *p* = 0.00053). Assays used all samples; Baseline *n* = 21; Follow Up DOAC *n* = 13; Follow Up LMWH *n* = 8.

**Table S1.** Olink inflammatory cytokine T48 panel‐ statistical analysis.

## Data Availability

Minimal anonymised data sets are available upon request.
