# Supplementary figures and images for: Direct Oral Anticoagulants Are Comparable to Low Molecular Weight Heparin at Sustaining the Circulating Extracellular Vesicle and Inflammatory Profiles of Cancer Associated Thrombosis Patients: An Observational Pilot Study
Source: Cancer Med. 2025 Apr 28;14(9):e70920. doi: 10.1002/cam4.70920 (PMC12035765; doi:10.1002/cam4.70920)

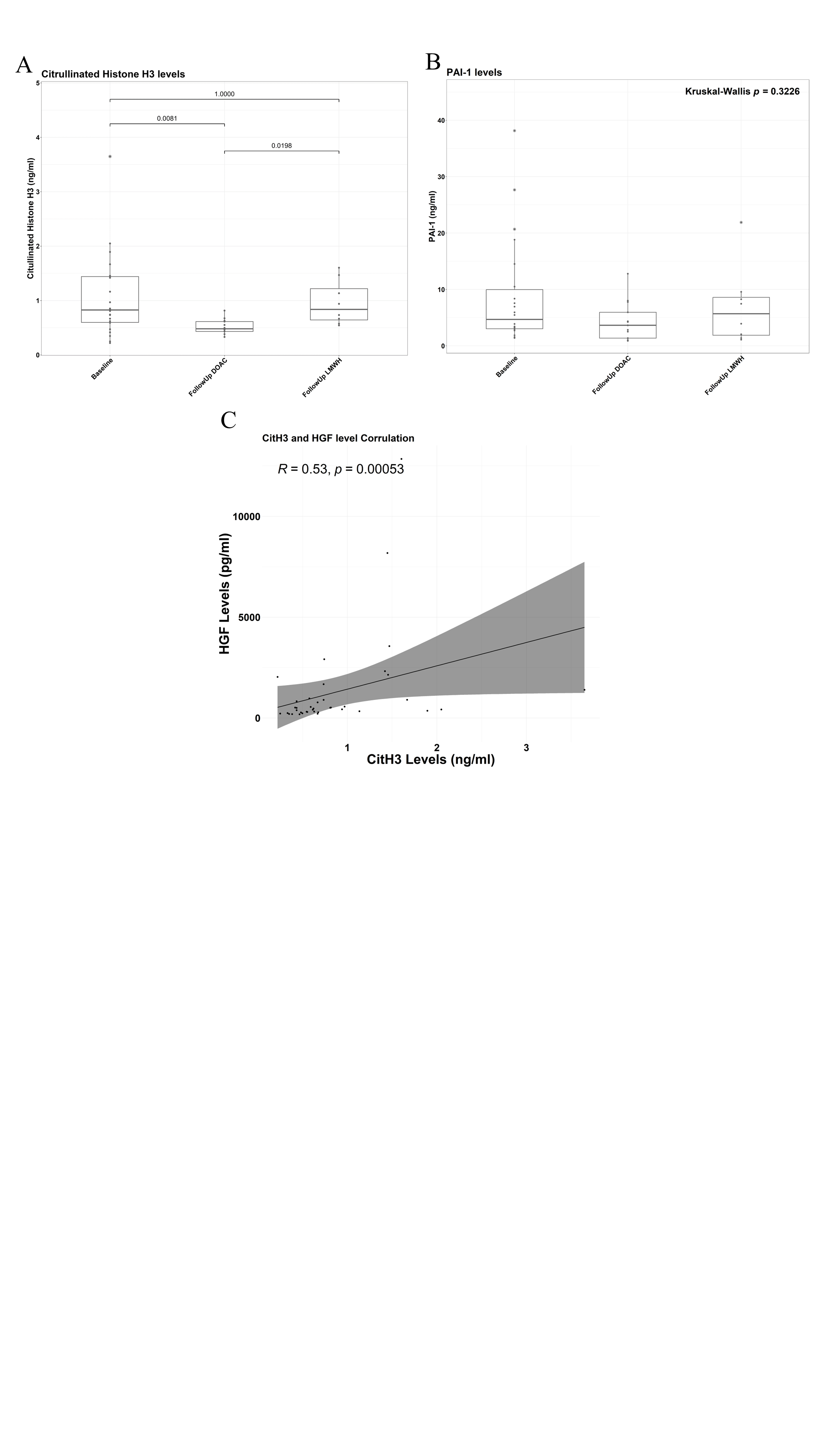

Supplement: Supplementary file 1 — Figure S1. Citrullinated histone H3 and PAI levels in CAT patients treated with DOACs compared to LMWH. Immunoassays quantified hypercoagulable markers of citrullinated histone H3 (CitH3) and PAI‐1 in PPP of CAT patients treated with DOAC compared to LMWH treatment. (A) Results show a significant reduction in CitH3 in the DOAC arm compared to both baseline and LMWH, however levels in the LMWH arm did not deviate from baseline (Kruskal–Wallis test, p = 0.009242). (B) PAI‐1 remained constant between both anticoagulant drugs (Kruskal–Wallis test, p = 0.3226). (C) A statistically significant moderate correlation was observed between CitH3 and the inflammatory protein HGF from the Olink analysis (R = 0.54, Spearman p = 0.00053). Assays used all samples; Baseline n = 21; Follow Up DOAC n = 13; Follow Up LMWH n = 8. [file CAM4-14-e70920-s002.tif]
