# Supplementary material for: Direct Oral Anticoagulants Are Comparable to Low Molecular Weight Heparin at Sustaining the Circulating Extracellular Vesicle and Inflammatory Profiles of Cancer Associated Thrombosis Patients: An Observational Pilot Study
Source: Cancer Med. 2025 Apr 28;14(9):e70920. doi: 10.1002/cam4.70920 (PMC12035765; doi:10.1002/cam4.70920)
Supplement: Supplementary file 2 — Table S1. Olink inflammatory cytokine T48 panel‐ statistical analysis. [file CAM4-14-e70920-s001.docx]

**Supplementary Table 1. Olink Inflammatory Cytokine T48 Panel- Statistical Analysis**

| **Protein ID** | **Baseline**  **(n=19; median ± IQR)** | **Follow Up DOAC (n=13; median ± IQR)** | **Follow Up LMWH (n=8; median ± IQR)** | **Missing Freq % (all 3 groups)** | **KW *p*** | **KW Benjamini Hochberg correction (multiple testing)** | **Threshold** |
| --- | --- | --- | --- | --- | --- | --- | --- |
| HGF | 671.6 (± 1466.75) | 300.76 (±167.65) | 655.46 (±1290.36) | 2% | 0.007447 | 0.2950 | Non-significant |
| FLT3LG | 88.46 (±94.41) | 170.64 (± 67.29) | 121.29 (±82.79) | 0% | 0.01311 | 0.2950 | Non-significant |
| IL27 | 8.13 (± 9.24) | 5.37 (±3.22) | 13.53 (±19.57) | 0% | 0.0493 | 0.5641 | Non-significant |
| IL10 | 9.58 (± 13.80) | 5.56 (±2.83) | 14.22 (±13.47) | 0% | 0.08706 | 0.5641 | Non-significant |
| IL17F | 0.496 (±0.74) | 0.276 (± 0.17) | 0.571 (± 0.67) | 37% | 0.0974 | 0.5641 | Non-significant |
| EGF | 27.59 (± 44.76) | 19.34 (±13.90) | 27.53 (± 24.08) | 0% | 0.09879 | 0.5641 | Non-significant |
| TGFA | 6.71 (± 5.61) | 5.11 (± 4.23) | 8.14 (± 2.14) | 0% | 0.1349 | 0.5641 | Non-significant |
| MMP12 | 277.32 (± 293.06) | 222.11 (± 177.00) | 527.80 (±301.99) | 0% | 0.1367 | 0.5641 | Non-significant |
| VEGFA | 298.11 (± 331.13) | 272.10 (± 152.67) | 420.19 (± 340.42) | 0% | 0.1458 | 0.5641 | Non-significant |
| CCL7 | 1.10 (± 0.93) | 0.862 (± 0.91) | 1.57 (± 1.08) | 0% | 0.1466 | 0.5641 | Non-significant |
| OSM | 2.09 (± 2.08) | 1.17 (±2.74) | 2.84 (± 2.49) | 0% | 0.1483 | 0.5641 | Non-significant |
| CCL3 | 7.79 (± 3.75) | 5.72 (± 3.92) | 10.02 (±3.69) | 0% | 0.1504 | 0.5641 | Non-significant |
| LTA | 5.37 (±1.42) | 6.30 (± 2.87) | 5.49 (± 1.36) | 0% | 0.2337 | 0.7154 | Non-significant |
| IL7 | 1.76 (± 1.32) | 1.031 (±0.58) | 1.18 (± 0.64) | 0% | 0.2378 | 0.7154 | Non-significant |
| TNFSF10 | 297.97 (± 97.84 | 319.80 (±95.33) | 379.58 (±188.82) | 0% | 0.2545 | 0.7154 | Non-significant |
| CSF1 | 175.91 (± 50.67) | 154.86 (± 26.46) | 174.67 (± 39.77) | 0% | 0.264 | 0.7154 | Non-significant |
| OLR1 | 51.04 (± 64.01) | 26.97 (±74.95) | 67.96 (± 21.26) | 0% | 0.2703 | 0.7154 | Non-significant |
| IL2 | 0.014 (±0.020) | 0.017 (±0.020) | 0.010 (± 0.012) | 91% | 0.2982 | 0.7456 | Non-significant |
| IL1B | 0.166 (±0.171) | 0.074 (± 0.127) | 0.149 (± 0.242) | 77% | 0.3926 | 0.8773 | Non-significant |
| CXCL8 | 10.59 (± 26.89) | 8.60 (± 15.19) | 14.78 (± 43.48) | 0% | 0.3933 | 0.8773 | Non-significant |
| CCL19 | 59.23 (±29.96) | 62.14 (±45.75) | 71.70 (±87.24) | 0% | 0.4123 | 0.8773 | Non-significant |
| TNF | 17.87 (±9.76) | 19.33 (±10.39) | 25.29 (±9.49) | 0% | 0.4964 | 0.8773 | Non-significant |
| CXCL12 | 122.59 (±46.80) | 99.71 (±46.94) | 119.87 (±46.91) | 0% | 0.5075 | 0.8773 | Non-significant |
| CCL8 | 31.59 (± 16.19) | 22.26 (±12.24) | 26.54 (±18.25) | 0% | 0.5117 | 0.8773 | Non-significant |
| IL13 | 0.251 (± 0.19) | 0.221 (± .03) | 0.258 (±0.25) | 74% | 0.5148 | 0.8773 | Non-significant |
| IL6 | 12.01 (±27.23) | 7.34 (± 9.47) | 8.87 (± 10.64) | 0% | 0.539 | 0.8773 | Non-significant |
| IFNG | 0.257 (± 0.19) | 0.434 (± 0.71) | 0.605 (± 0.578) | 5% | 0.5439 | 0.8773 | Non-significant |
| CCL2 | 396.39 (± 262.43) | 394.06 (±151.24) | 571.24 (299.38) | 0% | 0.5459 | 0.8773 | Non-significant |
| CSF2 | 0.191 (± 0.16) | 0.198 (± 0.10) | 0.263 (± 0.366) | 72% | 0.5821 | 0.8945 | Non-significant |
| IL17A | 0.518 (± 0.89) | 0.856 (± 0.83) | 0.701 (± 1.05) | 7% | 0.6132 | 0.8945 | Non-significant |
| CXCL11 | 52.67 (± 75.08) | 53.99 (± 53.22) | 43.93 (43.72) | 0% | 0.634 | 0.8945 | Non-significant |
| CSF3 | 155.54 (± 57.15) | 120.36 (± 101.53) | 137.32 (± 114.89) | 0% | 0.6361 | 0.8945 | Non-significant |
| CCL4 | 52.55 (± 30.84) | 43.98 (± 17.07) | 57.04 (± 37.10) | 0% | 0.6938 | 0.8997 | Non-significant |
| CXCL9 | 102.23 (± 82.74) | 114.80 (± 104.59) | 139.83 (± 120.39) | 0% | 0.699 | 0.8997 | Non-significant |
| IL17C | 36.46 (± 77.16) | 28.70 (± 31.25) | 42.82 (± 120.08) | 0% | 0.7298 | 0.8997 | Non-significant |
| TSLP | 0.028 (± 0.052) | 0.019 (± 0.070) | 0.028 (±0.07) | 93% | 0.7441 | 0.8997 | Non-significant |
| CCL13 | 48.76 (± 37.34) | 50.00 (± 20.94) | 63.99 (±39.23) | 0% | 0.7687 | 0.8997 | Non-significant |
| CXCL10 | 70.71 (± 86.80) | 93.10 (± 121.01) | 74.08 (± 113.52) | 0% | 0.7762 | 0.8997 | Non-significant |
| IL15 | 17.90 (± 15.06) | 15.71 (± 11.19) | 15.89 (±6.04) | 0% | 0.8304 | 0.8997 | Non-significant |
| IL18 | 447.48 (±386.15) | 329.13 (±93.99) | 359.05 (± 256.14) | 0% | 0.8602 | 0.8997 | Non-significant |
| TNFSF12 | 361.91 (± 182.81) | 324.47 (±129.57) | 400.45 (±350.13) | 0% | 0.8658 | 0.8997 | Non-significant |
| IL4 | 0.063 (± 0.013) | 0.0681 (±0.007) | 0.0625 (±0.016) | 91% | 0.8744 | 0.8997 | Non-significant |
| CCL11 | 72.12 (± 67.84) | 73.96 (± 49.06) | 87.10 (± 37.77) | 0% | 0.8891 | 0.8997 | Non-significant |
| MMP1 | 687.08 (± 797.23) | 629.15 (1019.08) | 727.24 (± 537.59) | 0% | 0.8979 | 0.8997 | Non-significant |
| IL33 | 0.182 (± 0.163) | 0.165 (±0.129) | 0.191 (±0.103) | 91% | 0.8997 | 0.8997 | Non-significant |
